# Recent advances in analysis of capsaicin and its effects on metabolic pathways by mass spectrometry

**DOI:** 10.3389/fnut.2023.1227517

**Published:** 2023-07-28

**Authors:** Zifang Peng, Wenfen Zhang, Xu Zhang, Jian Mao, Qidong Zhang, Wuduo Zhao, Shusheng Zhang, Jianping Xie

**Affiliations:** ^1^College of Chemistry, Zhengzhou University, Zhengzhou, Henan, China; ^2^Zhengzhou Tobacco Research Institute of CNTC, Zhengzhou, Henan, China; ^3^School of Ecology and Environment, Zhengzhou University, Zhengzhou, Henan, China; ^4^Food Laboratory of Zhongyuan, Flavor Science Research Center of Zhengzhou University, Luohe, Henan, China

**Keywords:** capsaicin, mass spectrometry, food analysis, metabolic pathways, biological activities

## Abstract

Capsaicin is the main food active component in *Capsicum* that has gained considerable attention due to its broad biological activities, including antioxidation, anti-inflammation, anti-tumor, weight regulation, cardiac protection, anti-calculi, and diurnal-circadian regulation. The potent biological effects of capsaicin are intimately related to metabolic pathways such as lipid metabolism, energy metabolism, and antioxidant stress. Mass spectrometry (MS) has emerged as an effective tool for deciphering the mechanisms underlying capsaicin metabolism and its biological impacts. However, it remains challenging to accurately identify and quantify capsaicin and its self-metabolites in complex food and biological samples, and to integrate multi-omics data generated from MS. In this work, we summarized recent advances in the detection of capsaicin and its self-metabolites using MS and discussed the relevant MS-based studies of metabolic pathways. Furthermore, we discussed current issues and future directions in this field. In-depth studies of capsaicin metabolism and its physiological functions based on MS is anticipated to yield new insights and methods for preventing and treating a wide range of diseases.

## Introduction

*Capsicum* is primarily used as a food ingredient and is appreciated for its unique pungent taste, aroma, and color. This plant is also a rich source of nutrients, such as vitamin C, beta-carotene, calcium, iron, and potassium, among others, which can improve immunity, blood circulation, and vision (1). Due to its high nutritional value and distinctive flavor, the consumption of *Capsicum* has continuously increased over the years. In 2020, the global production of *Capsicum* reached 535 thousand tons, reflecting the growing demand for this versatile and beneficial plant (2). Capsaicin and its related compounds, such as dihydrocapsaicin and *Capsicum* carboxamide, are predominantly found in *Capsicum*. These capsaicinoids are widely distributed in the fruits and seeds of *Capsicum* and are responsible for the characteristic pungent flavor of this plant (3). The investigation of capsaicin metabolism has attracted considerable attention due to its potential effect on various physiological functions. Capsaicin is primarily metabolized in the liver and intestine of the human body (4). Upon entering the body, esterases in the intestine hydrolyze capsaicin into free capsaicin and capsaicin ester (5). These compounds then enter the blood circulation and generate various metabolites, including hydroxylates, amides, sulfates among others (6). These metabolites play roles in different physiological activities by regulating several signaling pathways and biological processes *in vivo*, such as cell growth, apoptosis, differentiation, and immune response (7–10). With the development of metabolomics and biotechnology, several studies have investigated the role of capsaicin metabolism and its products on human health (11–18). Recent research has demonstrated that capsaicin and its self-metabolites possess diverse biological activities, including antioxidation, anti-inflammatory, anti-tumor, weight regulation, cardiac protection, anti-calculus, and circadian regulation properties (11–15). These functions are accomplished through the modulation of various metabolic pathways including lipid metabolism, energy metabolism, and antioxidant stress, among other pathways (16–18). Thus, exploring the metabolism and physiological functions of capsaicin is of great interest, as it may offer novel insights into the prevention and treatment of numerous diseases.

The detection and identification of capsaicin and its metabolites in food and biological samples are of great importance due to their physiological significance. Numerous traditional methods have been used to detect metabolites. Among them are nuclear magnetic resonance (NMR) techniques, which offer the advantage of providing structural information on compounds without the need for sample separation and purification (19). However, in analysis of complex biological samples, NMR techniques are relatively insensitive and time-consuming. Other techniques, such as infrared spectroscopy (IR) and capillary electrophoresis (CE), are also useful for detecting and analyzing capsaicin metabolism (20, 21). While IR spectroscopy can provide information on chemical bonds, it cannot discern the three-dimensional structure of molecules. CE is a separation technique that has high separation efficiency and requires minimal amount of sample. However, the loss and degradation of metabolites are potential issues during sample pretreatment and manipulation. MS is an effective analytical technique that can be used for investigating the composition and dynamics of metabolites in living organisms. This technology has high-throughput capability in detection and quantification of metabolites in biological samples, such as blood, urine, and tissues, thus is beneficial for the exploration of the interactions between metabolites, changes in metabolic pathways, and their association with diseases (22–25). MS offers numerous advantages, including high sensitivity, high resolution, high specificity, and high throughput; thus, it can cover a wide range of metabolites, such as small molecule, lipid, carbohydrate, and nucleotide metabolites (26, 27). It also facilitates the quantitative analysis of metabolites, allowing for the accurate measurement of metabolite concentration and change trends, as well as the accurate identification of metabolite structures and types. MS is widely used to detect capsaicin and its self-metabolites in various types of metabolomics research, including lipid metabolomics, energy metabolomics, and proteomics (28). With the continuous development of MS technology and the establishment of MS libraries, as well as the ongoing refinement of data analysis methods, MS will likely be used more extensively in the study of capsaicin metabolism and other related metabolisms.

Here, we first summarized the recent progress in MS-based detection of capsaicin and its self-metabolites. In addition, considering the abundant physiological activities of capsaicin and its metabolites in organisms, we discussed MS-based studies of effects of capsaicin on various metabolic pathways in organisms. Finally, challenges and perspectives of MS-based studies of capsaicin metabolism and related metabolisms are presented. This review article highlights the critical role of MS in the research of capsaicin and deepens our understanding of the metabolic process of capsaicin in the human body, thereby clarifying the connection between capsaicin and health.

## MS-based analysis of capsaicin and its metabolites

The applications of MS in detecting capsaicin and its metabolites are broadened due to the continuous improvement in the technique (Table 1), such as gas chromatography-mass spectrometry (GC-MS) for better sensitivity, liquid chromatography-tandem mass spectrometry (LC-MS/MS) for expanding the application types of samples, matrix-assisted laser desorption ionization time-of-flight mass spectrometry (MALDI-TOF-MS) for user-friendly operations, high efficiency for defectively detected MS techniques.

**Table 1:** MS-based determinations of capsaicin in food and biological matrix.

Analytical method	Matrix	Analyte	Reference
GC-MS	Chili powder, chili sauce, and chili-flavored potato chips	Capsaicin	(30)
GC-qMS	Five different types of *Capsicum*	Capsaicin, dihydrocapsaicin, phenolic acids	(31)
GC-qMS, GC-TOF-MS, GC-FID	*Capsicum* at different ripening stages	Capsaicins, amino acids, organic acids, sugars, sugar alcohols, free phenolic acids, carotenes, phytosterols, polyphenols, tocopherols, and volatile fatty acids	(33)
LC-MS/MS	Human and porcine hepatocyte components and a human lung cancer cell line (A549)	Capsaicin, dihydrocapsaicin and their metabolites	(34)
UPLC Q-Orbitrap HRMS/MS	Human liver microsomes	19 phase I metabolites and 13 GSH complexes of capsaicin	(35)
UHP-MS/MS	Gutter oil	Capsaicin, dihydrocapsaicin and n-vaninoamide	(36)
DART-TOF-MS	46 varieties of *Capsicum* and their seeds	Capsaicin	(37)
MALDI-MS	Perfume and herbs	Capsaicin	(38)
IR-MALDI-MS	Mouse brain and *Danaus plexippus* caterpillar sections	Capsaicin, dihydrocapsaicin and their metabolites	(39)
TD-CFI-MS	Chips, crisps, spicy peanuts, spicy vicia faba, spicy kelp silk, chili powder, sesame paste, peanut butter, sunflower seed oil, capsicol, mushroom sauce, medical tape, Guanjie zhitonggao patch, Shexiang zhentonggao patch, Lajiao fengshigao patch, and Shidishui tincture	Capsaicin	(40)

### GC-MS

GC-MS is an analytical technique popularly used for the detection of capsaicin and its self-metabolites. The process begins with sample separation using GC, where the sample is vaporized and separated into compounds (29). Within the GC-MS, the compounds are divided into ions, and their mass are recorded.

GC-MS is an effective method that can detect capsaicin in plant samples. For example, using GC-MS technique, Jaroslav and his colleagues determined the amount and type of capsaicin presented in chili powder, chili sauce, and chili-flavored potato chips (30). The processing of sample only requires 30 min prior to GC-MS analysis. This new method does not require significant amounts of organic solvents during extraction and purification steps, as main capsaicin is directly derivatized with hexamethyldisilazane. The linear calibration ranges of this new method are between 0.025–810 μg mL^−1^ and 0.04–440 μg mL^−1^. The derivatization efficiency of capsaicin and dihydrocapsaicin is 100% after 30 min of reaction. Through this method, capsaicin and dihydrocapsaicin were successfully extracted from chili powder, chili sauce, and potato chips. The contents of capsaicin and dihydrocapsaicin in 25 analyzed samples were 0.00014 to 9.86% and from 0.00022 to 3.45%, respectively. The outcomes obtained through this approach are in agreement with those acquired using the established AOAC method, which confirms the applicability of the approach. Kim et al. employed GC-quadrupole-MS (GC-qMS) to identify and quantify metabolites of capsaicin and phenolic acids (31). They detected two capsaicins and five phenolic acids in five different types of *Capsicum*. Capsaicin in *Capsicum* was found to be approximately 2.5 times more potent than dihydrocapsaicin, which is consistent with a previous study (32). The authors identified the presence of three types of phenolic acids, namely *p*-hydroxybenzoic acid, vanillic acid, and *p*-coumaric acid, in green peppers and detected sinapic acid and ferulic acid in *Capsicum*. They also discovered that the levels of these capsaicin metabolites increased as the *Capsicum* matured. Kim et al. developed a high-throughput platform with integrated GC-time-of-flight (TOF)-MS, GC-qMS, and GC-flame ionization detector (FID) for the metabolic analysis of phytochemicals in *Capsicum* at different ripening stages (33). They conducted comprehensive analysis of various compound including capsaicins, organic acids, amino acids, free phenolic acids, volatile fatty acids, sugars, sugar alcohols, phytosterols, carotenes, polyphenols, and tocopherols. The study revealed a significant correlation between metabolic changes of *Capsicum* and maturity and spiciness of fruits. These findings contribute significantly to the metabolomics of *Capsicum* cultivars and are valuable for the breeding of new varieties. Despite the enhanced coverage of metabolite detection that the integrated GC-MS technologies have achieved, the detection of non-volatile and polar metabolites remains challenging.

### LC-MS

LC-MS is a highly effective analytical technique for detecting and analyzing capsaicin and its self-metabolites. The method involves the use of LC to separate the sample, followed by MS to detect and quantify the individual components that have been separated. Capsaicin and its self-metabolites are ionized by MS and separated according to their mass charge ratios. The abundances of the detected and recorded ionized components can be used to identify and quantify capsaicin and its metabolites in complex samples.

LC-MS is particularly useful for the detection of non-volatile and polar metabolites, which cannot be detected by GC-MS. It also has high sensitivity, selectivity, and accuracy, which make it a powerful tool for the analysis of complex samples, including biological fluids and tissues. Halme et al. compared the *in vitro* metabolism and cytotoxicity of capsaicin and dihydrocapsaicin to human and porcine hepatocytes and a human lung cancer cell line (A549) (34). LC-MS/MS was utilized to identify and analyze the metabolites after centrifugation and deproteinization. They were able to detect a new aliphatic hydroxyl metabolite associated with dihydrocapsaicin and a new I phase metabolite of capsaicin in the hepatocytes. Additionally, two new conjugates, a glycine conjugate and a double glutathione (GSH) conjugate, were identified as capsaicin and dihydrocapsaicin metabolites. Capsaicin metabolites in various forms, such as ω-hydroxylated and alkyl dehydrogenated forms, as well as the glycine conjugate of capsaicin, were also detected in A549 cells. In this study, the LC-MS/MS method was used to screen and identify the metabolites of capsaicin and dihydrocapsaicin. However, due to limited LC-MS database, finding new small molecule metabolites by a single LC-MS technique is difficult. Qin et al. thoroughly screened and identified capsaicin metabolites in human liver microsomes using UPLC Q-Orbitrap HRMS/MS combined with post-acquisition data mining tools (35). This method overcomes the difficulty of discovering novel metabolites and enables rapid examination and determination of metabolites. The study identified nineteen phase I metabolites and thirteen glutathione (GSH) complexes of capsaicin, and summarized an overview of their metabolic pathways. The findings suggest that capsaicin can undergo single or multiple metabolic pathways to produce various metabolites, providing insights into the transformation process of capsaicin *in vivo*. In addition to the ability to accurately determine capsaicin and metabolites in biological samples, HPLC-MS also has a wide range of applications in environmental samples. Lu et al. extracted capsaicin and its analogues from gutter oil using magnetic solid-phase extraction (MSPE), and determined capsaicin, dihydrocapsaicin and n-vaninoamide from gutter oil using UHPLC-MS/MS (UHP-MS/MS) (36). Under optimized preconditioning conditions, the method has the limit of detection (LOD) and limit of quantification (LOQ) of 0.15 and 0.4 μg L^−1^, respectively. In the detection of all three compounds, the linear range was 0.4–200 μg L^−1^ and the correlation coefficients (*R*^2^) exceeded 0.996. In spike recovery experiments the recoveries of capsaicin and its analogues were between 71.2 and 110.6%. LC-MS can accurately measure trace amounts of capsaicin and its self-metabolites in samples due to its extremely high sensitivity. However, the whole separation procedure is time-consuming and requires the use of a large amount of solvents and high flow rates.

### Direct analysis-based MS

Various direct detection techniques without separation and extraction have been employed to rapidly identify capsaicin and its metabolites. One such technique is direct analysis in real-time time-of-flight mass spectrometry (DART-TOF-MS), an advanced direct MS technique that enables rapid examination of a wide variety of samples without the need of pre-processing steps to rapidly achieve accurate analysis results. With real-time monitoring capabilities, DART-TOF-MS technology can track changes in compound contents of samples in real-time, thus is highly suitable for online detection and process monitoring. Due to its superior efficiency and accuracy, as well as time-saving capabilities, DART-TOF-MS technique has been successfully applied to detect capsaicin in food. Tobolka and colleagues employed DART-TOF-MS technique to quantify the capsaicin content in 46 varieties of *Capsicum* and their seeds (37). This newly developed method was compared with the diode array detection (HPLC-DAD) method. The study found that in detection of capsaicin content in pepper fruit and seed, the correlation coefficient of the DART-TOF-MS method and the HPLC-DAD method was 0.845 and 0.776, respectively. DART-TOF-MS is a rapid and reliable technique that can distinguish between different types of *Capsicum* and their seeds based on their spiciness and capsaicin contents. MALDI-MS/SALDI- MS is an MS-based method that has been widely adopted in the swift detection and analysis of capsaicin, as well as in the determination of its structure and composition. This technique involves using a laser to illuminate a complex between matrix and sample. The matrix absorbs the energy from the laser and produces high-energy photons that then transform molecules in the sample into ions. These ions are subsequently introduced into a mass spectrometer for detection and analysis. Li et al. used Co-NC as an adsorbent of capsaicin and a substrate for SALDI-MS in the rapid detection of capsaicin (38). To improve the sensitivity of MALDI-MS to capsaicin, Schneemann et al. developed a new method called IR-MALDI-MS (39). The signals of capsaicin, dihydrocapsaicin and their metabolites in plasma were found to increase by 1,000 times. Matrix effect, which refers to the influence of compounds in the matrix on the mass spectrum signal of the substance being detected, is a common problem in MS. Matrix effect can lead to changes in intensity and shape of signal peaks, which can in turn affect the accuracy and repeatability of analysis results. To mitigate the impact of matrix effects in quantitative analysis of intricate samples, Zhang et al. devised thermal desorption carbon fiber ionization MS (TD-CFI-MS) (40). In this TD-CFI-MS technique, after a liquid or solid sample comes into contact with the high-temperature surface of a cermet heater (MCH), the analytes in the sample are instantaneously desorbed and carried as gas to the ionization zone. The ionization region contains an abundance of protonated water mass [(H_2_O)_n_H^+^] generated by a carbon fiber ion source. The gas-phase analytes are ionized after interacting with evaporating protonated water clusters; and subsequently, the resulting analyte ions are transferred to a mass spectrometer for further analysis. TD-CFI-MS has been successfully applied to quantitatively analyze capsaicin in various real samples, and the matrix effect was found to range between 93.3 and 97.6%, an indication that the method can effectively reduce the influence of matrix effect and provide a straightforward and rapid analysis for product quality control.

## MS-based studies of capsaicin’s effects on metabolic pathways

Capsaicin possesses a wide range of biological activities *in vivo*, including antioxidation, anti-inflammatory, anti-tumor, regulation of blood sugar and lipid and other biological activities (13–15). It also affects various complex metabolic pathways, including inflammation, apoptosis, cell signal transduction, lipid metabolism, and glucose metabolism, among other pathways (16–18). Recent studies on MS have shown that capsaicin has broad effects on the metabolic pathways of organisms (Table 2). MS can be used to quantify and analyze the metabolites of capsaicin *in vivo*, deeply understanding the mechanism by which capsaicin influences the metabolism of organisms.

**Table 2:** MS-based studies of capsaicin’s effects on metabolic pathways.

Study design	Metabolic pathway	Analytical method	Results	Reference
RAW264.7 cells	Inflammatory metabolic pathway	LC-MS /MS	↓PKM2, LDHA, COX-2	(41)
Colon tissues of mice	Inflammatory metabolic pathway	DDA-MS	More than 50 proteins were differentially expressed	(42)
Mice fed with beef fat	Lipid metabolic pathway	GC-MS	↓TC, TG, LDL-C ↑HDL-C	(43)
Sprague Dawley rats	Lipid metabolic pathway	HPLC-TOF-MS	↓TC, TG ↑CDCA, DCA, β-MCA, T-β-β-MCA	(44)
Blood and stool samples	Energy metabolic pathway	LC-MS/MS	↑n-oleylethanolamine, n-linoleylethanolamine, 2-oleylglycerol, n-acylethanolamine, n-docosahexaenoethanolamine, n-docosahexaenoethanolamine	(45)
Human intestinal epithelial cells	Energy metabolic pathway	MALDI-TOF-MS	↑Glycosylation enzymes, PGM, TPI	(46)
Blood	Oxidative stress pathway	UHPLC-Q-TOF-MS	↓Cholic acid, pipecolic acid, taurine, pyroglutamic acid, gamma-glutamylcysteines ↑Deoxycholic acid, Carnosine	(48)
Rat epididymal white adipose tissue	Oxidative stress pathway	MALDI-TOF-MS	↓Flavoprotein, myosin light chain, beta 1 globulin, EF-2 and malate dehydrogenase, aldo-keto reductase ↑Flavoprotein, beta 1 globulin, myosin light chain, malate dehydrogenase, coiled-coil domain containing like, Olr1434, aldehyde reductase, aldo-keto reductase 1, EF-2, E3 ubiquitin protein ligase	(47)
Mice	Intestinal flora metabolic pathway	LC-MS	↑TβMCA, TωMCA ↓CA, TCA	(49)
Mice	Intestinal flora metabolic pathway	GC-MS	↑Serum levels of substance P, calcitonin gene-related peptide, SCFAs	(50)

↑, concentration up-regulation; ↓, concentration down-regulation.

### Effects of capsaicin on inflammatory-related metabolic pathway

Capsaicin exerts its anti-inflammatory effects by inhibiting the expression of genes or proteins associated with inflammation, as well as inhibiting the inflammatory response. Through the use of LC-MS/MS, Zhang et al. confirmed that pyruvate kinase isoenzyme type M (PKM), L-lactate dehydrogenase A (LDHA) and prostaglandin G/H synthase 2 (COX-2) are the target proteins of capsaicin in RAW264.7 cells (41). Capsaicin inhibits the Warburg effect by binding directly to Cys424 residue and LDHA of pyruvate kinase isoenzyme type M2 (PKM2). In addition, capsaicin targets COX-2 and down-regulates its expression, which results in the further inhibition of inflammation. The current findings suggest that capsaicin reduces the inflammatory response and the Warburg effect in sepsis by targeting PKM2-LDHA and COX-2, independently of the TRPV1 receptor. Therefore, capsaicin may be used as a new active ingredient to treat sepsis and inflammation. Chen and colleagues gathered proteomic data in the colon tissues of capsaicin-treated mice with chronic colitis (42). A total of 2,763 proteins were detected using MS, among which several differentially expressed proteins were found closely associated with inflammatory pathways. The proteomic data revealed that more than 50 proteins were differentially expressed. A comprehensive analysis of proteomic and transcriptional data revealed the key pathways by which capsaicin exacerbates colitis recurrence and the effect of neutrophils on intestinal inflammation. Overall, the MS method can be used to identify proteins, enzymes and metabolites associated with inflammation and to study the effect of capsaicin on inflammatory metabolism.

### Effects of capsaicin on lipid metabolic pathway

Capsaicin regulates lipid metabolism by stimulating fat breakdown and oxidation and inhibiting fat synthesis and assimilation. Li et al. investigated the effects of capsaicin on lipid metabolism using rodents by feeding them with beef fat for 12 weeks and then analyzing the levels of various lipid markers in their serum (43). Using GC-MS analysis, they showed that mice fed with beef fat containing capsaicin had lower body weight, fat mass, and abdominal fat index compared to those fed with beef fat without capsaicin. Furthermore, the serum levels of total cholesterol (TC), triglycerides (TG), and low-density lipoprotein cholesterol (LDL-C) in mice fed with beef fat containing capsaicin were significantly reduced compared to those in mice fed beef fat without capsaicin. Capsaicin was also found to prevent the decrease in high-density lipoprotein cholesterol (HDL-C) levels in serum of rodents fed with a high-fat diet. These results suggest that capsaicin plays a crucial role in regulating fat generation, transfer, and differentiation in mice fed with a high beef fat diet by maintaining lipid homeostasis. Gong et al. studied the mechanism of capsaicin activity of gut microbes and bile acids (BA) (44). They employed MS to examine BA in Sprague Dawley (SD) rats. Their results showed that capsaicin increased the levels of oxycholic acid (CDCA), deoxycholic acid (DCA), β- muricholic acid (β-MCA), and tauro-β-muricholic acid sodium taurine (T-β-β-MCA), which are compounds involved in modulating Fgf15 inhibition by the Farnesoid X receptor (FXR). Capsaicin also increased the expression of CYP7A1, resulting the reduction of triglyceride (TG) and total cholesterol (TC). Overall, the MS analysis of lipid metabolites in serum and tissues show that capsaicin affects the expression of several key enzymes in lipid metabolic pathways, such as fatty acid synthetase and fatty acid oxidase, and fat distribution (e.g., by transferring fat from the abdomen to the liver). Accordingly, MS can provide an important means for studying the impact of capsaicin on lipid metabolism and revealing its mechanism of action in disease prevention and therapy.

### Effects of capsaicin on energy metabolic pathway

Studies have shown that capsaicin affects energy metabolism by regulating fatty acid metabolism and sugar metabolism. Consumption of capsaicin has been shown to promote calorie expenditure and fat oxidation, which can help in reducing weight and preventing obesity-related diseases. Capsaicin regulates the metabolic pathways by activating transcription factor and kinase signaling pathways. For example, capsaicin activates adenylate-activated protein kinase (AMPK) and protein kinase A (PKA), in turn enhancing the activity of the mitochondrial respiratory chain and promoting fatty acid oxidation. In addition, capsaicin can inhibit the activity of fatty acid synthetase, resulting in reduced fatty acid synthesis and limited fat deposition. In a study conducted by Claudia et al., a group of overweight/obese women of reproductive age were subjected to a 500 kcal d^−1^ restriction for 12 weeks while taking either capsaicin or a placebo (45). To evaluate the effects of the intervention, pre- and post-intervention blood and stool samples were subjected to LC-MS/MS, wherein the plasma levels of eCBome mediators and 16S metagenomic sequences in fecal microbiome taxa were analyzed. The *Capsicum* extract (CAE) used in the study was found to block the reduction of anti-inflammatory effects of endocrine mediators caused by reduced caloric intake, including TRPV1, GPR119 and/or peroxisome proliferator-activated receptor α (PPARα) agonists (*N*-oleyl-ethanolamine, *N*-linoleoyl-ethanolamine, 2-oleylglycerol, *N*-acylethanolamine and *N*-docosahexaenoethanolamine). The results of the study indicate that the inclusion of dietary capsaicin (administered in the form of CAE capsules) in a low-calorie intervention can impact the perception of appetite, energy intake and expenditure, as well as weight and fat distribution in physically active, overweight or obese women of reproductive age. In terms of sugar metabolism, capsaicin can promote insulin secretion, increase sugar utilization, and inhibit glucose absorption. All these effects help to maintain blood sugar balance and prevent the development of diabetes and other metabolic diseases. Han et al. investigated the mechanism underlying capsaicin-induced energy metabolism in human intestinal epithelial cells (Caco-2) using proteomics, real-time PCR, ATP measurement, and MTT data (46). The study revealed that capsaicin treatment led to the upregulation of glycosylation enzymes, phosphoglycerate mutase (PGM), and triosephosphate isomerase (TPI). Moreover, the treatment resulted in increased intracellular ATP levels (the end product of glycolysis), which suggests that capsaicin enhances the energy metabolism of Caco-2 by activating glycolytic enzymes. These findings provide insights into the mechanism of how capsaicin induces energy metabolism in Caco-2. MS has been widely used to study the effect of capsaicin on energy metabolism. MS can be employed to study the effect of capsaicin on energy metabolism of cells and tissues by using it to analyze proteins, metabolites, nucleic acids and other molecules in the metabolic pathways. For example, MS can be used to identify the activation of metabolic enzymes by capsaicin in human intestinal epithelial cells to enhance energy metabolism (46). The effect of capsaicin on lipids in liver and adipose tissue can also be studied by mass spectrometry, and the loss or increase of fat is associated with the TCA cycle. In this way, the effects of capsaicin on energy metabolism can also be indirectly understood (47). At present, MS has been widely used to study the effects of capsaicin on energy metabolism, as well as to provide strong support for further study of its biological effects.

### Effects of capsaicin on oxidative stress pathway

Changes to the internal and external environment of cells can lead to a biological response known as oxidative stress, resulting in the production of free radicals and oxides that damage various vital biological molecules such as DNA, proteins, and lipids. This can then lead to cell and tissue damage and malfunction. Capsaicin can mitigate the negative effects of oxidative stress on human health by scavenging these free radicals and reducing the oxidative stress response. In addition, capsaicin can reduce oxidative stress-induced cell damage by activating several signaling pathways and transcription factors, promoting the cell self-protection mechanisms, and enhancing the antioxidant capacity of cells. Using untargeted metabolomics, Xiao et al. demonstrated that capsaicin can control cellular oxidative stress (48). In their studies, ultra-high performance liquid chromatography-quadrupole time-of-flight MS (UHPLC-Q-TOF-MS) was used to detect differential metabolites associated with oxidative stress in plasma, including pipecolic acid, cholic acid, carnosine, taurine, deoxycholic acid, pyroglutamic acid, 3-indole-propionic acid, and gamma-glutamylcysteine. Interestingly, they found that pipertinic acid, a lysine metabolite, induced oxidative stress in the cerebral cortex of rats, while carnosine and taurine was found to exhibit antioxidant activity. Gamma-glutamylcysteine, pyroglutamate, and taurine were also found to be involved in the regulation of oxidative stress, likely due to its interaction with glutathione. These results suggest that capsaicin may play a role in regulating oxidative stress by altering metabolites involved in the process. In previous studies, the combination of two-dimensional electrophoresis with MALDI-TOF-MS was utilized for differential protease analysis of rat epididymal white adipose tissue. The findings revealed that the majority of identified proteins were associated with lipid metabolism and the regulation of oxidative stress (47). These studies demonstrate MS can be broadly applied to study the effects of capsaicin on oxidative stress.

### Effects of capsaicin on intestinal flora metabolic pathway

Capsaicin impacts the metabolism of intestinal flora through various ways, including regulating the relative abundance of flora, changing the metabolic pathways and regulating the activity of related metabolic enzymes. Capsaicin can also affect the overall health of hosts by influencing the intestinal floral metabolism involved in intestinal immune regulation, energy metabolism, and nervous regulation. Hui et al. employed LC-MS to determine the content of tauro-β-muricholic acid (TβMCA, the most effective natural ligand of the FXR) and taurine-conjugated CA (TCA, an agonist of FXR) (49). Following capsaicin treatment, conjugated BA/unconjugated BA ratio was found elevated in capsaicin-fed mice, along with an increase in TβMCA levels by approximate 1.5 folds. A heat map of the Pearson correlation between intestinal flora genera and BA indicated that lactic acid bacteria were negatively correlated with FXR antagonists (TβMCA, TωMCA) and FXR antagonist/agonist ratio, but positively correlated with agonists (CA, TCA). Other intestinal flora in the genera such as *Bacteroides*, *Parabacteroides* and *Parasutterella* were also found significantly associated with fecal taurine-conjugated BA. Thus, capsaicin enhanced the accumulation of BA conjugated, specifically FXR antagonist TβMCA, by reshaping the gut flora, which further affected FXR activity. Experiments proving that the intake of capsaicin can regulate the metabolism of intestinal microbes and has a certain physiological effect have also been carried out. Using MS, Xiang et al. investigated the dose-dependent effects of capsaicin on gastrointestinal health by administering mice with capsaicin at various doses of 40, 60, and 80 mg kg^−1^ (50). The findings showed that 40 mg kg^−1^ capsaicin had no adverse effects on the gastrointestinal tissue, while 60 mg kg^−1^ capsaicin caused severe inflammation of the jejunum, ileum, and colon, leading to gastrointestinal tissue damage. The findings also showed that capsaicin increased the serum levels of substance P and calcitonin gene-related peptide in a dose-dependent manner. SCFAs in the serum of mice given 80 mg kg^−1^ capsaicin were also found to drastically increase. The correlation analysis further revealed that the underlying mechanism might be attributed to the regulation of the gut microbiome, particularly for those in the genera *Bifidobacterium*, *Lactobacillus*, and *Butyricimonas*. Therefore, mice orally given 60 and 80 mg kg^−1^ capsaicin developed intestinal inflammation and had elevated serum neuropeptide and SCFA levels, likely due to the alterations in the gut microbiome. In conclusion, MS is a high-throughput and highly sensitive analytical technique that can be used to study the effect of capsaicin on intestinal flora. Using MS, the effects of capsaicin on different types of bacteria and the specific regulatory mechanisms, such as the inhibition of the bacterial growth or the increase of bacterial diversity, can be determined. The knowledge obtained from MS can contribute to a deeper understanding of capsaicin’s impacts on human health and can lay the groundwork for the prevention and therapy of enteric-related diseases.

## Challenges and future perspectives

The use of MS to examine capsaicin metabolism and its impact on biological metabolism is an active and growing area of research. The effects of capsaicin on metabolism and other physiological systems, such as energy metabolism, oxidative stress, and inflammation, have been the focus of a growing body of study over the past decade. MS is crucial in this type of research because it enables the identification and quantification of capsaicin and its self-metabolites in biological materials. It also helps to provide insights into the metabolic pathways involved in capsaicin metabolism and how capsaicin impacts metabolism. However, the metabolic pathways in capsaicin metabolism involve many enzymes and metabolic intermediates with low contents. Thus, studying these pathways can be challenging. Because MS’s ability to accurately identify and quantify metabolites is limited, more sensitive analytical techniques and specialized software that can analyze large volumes of data should be developed. Furthermore, the effects of capsaicin on organism metabolism are complex and can be influenced by a variety of factors, such as individual variations and environmental conditions, which may impact the reproducibility and accuracy of the results. Differences in capsaicin metabolism among individuals and groups also make it challenging to draw consistent conclusions in investigations, and this highlights the importance of large-scale research to fully understand the influence of capsaicin on cellular metabolism. Despite these obstacles, the ongoing development and enhancement of MS methods should provide additional opportunities to study the effects of capsaicin on metabolism. For instance, single-cell MS may be used to investigate the metabolism of capsaicin in various cell types and its impact on cell function. Additionally, MS could be used to provide insights into the tissue-level metabolic dynamics of capsaicin and reveal its *in vivo* mode of action. Finally, other high-throughput techniques, such as genomics, transcriptomics, and proteomics, could be used to implement MS in the investigation of capsaicin’s metabolic pathways and regulatory mechanisms.

## Author contributions

ZP and WZ wrote the original draft of the manuscript. XZ, JM, QZ and WZ conducted the searching processes. SZ reviewed and edited the manuscript. JX supervised the manuscript and did the funding acquisition. All authors contributed to the article and approved the submitted version.

## Funding

Financial supports from the National Natural Science Foundation of China (21140031), China Postdoctoral Science Foundation (2022 M710167) and Postdoctoral Research Grant in Henan Province (202102080) are gratefully acknowledged.

## Conflict of interest

The authors declare that the research was conducted in the absence of any commercial or financial relationships that could be construed as a potential conflict of interest.

## Publisher’s note

All claims expressed in this article are solely those of the authors and do not necessarily represent those of their affiliated organizations, or those of the publisher, the editors and the reviewers. Any product that may be evaluated in this article, or claim that may be made by its manufacturer, is not guaranteed or endorsed by the publisher.
